# Sociodemographic factors associated with vaccine hesitancy in the South Asian community in Canada

**DOI:** 10.17269/s41997-024-00885-7

**Published:** 2024-05-07

**Authors:** Baanu Manoharan, Rosain Stennett, Russell J. de Souza, Shrikant I. Bangdiwala, Dipika Desai, Sujane Kandasamy, Farah Khan, Zainab Khan, Scott A. Lear, Lawrence Loh, Rochelle Nocos, Karleen M. Schulze, Gita Wahi, Sonia S. Anand

**Affiliations:** 1 Department of Health Research Methods, Evidence and Impact, McMaster University, Hamilton, ON Canada; 2 Population Health Research Institute, Hamilton, ON Canada; 3 Faculty of Health Sciences, Simon Fraser University, Burnaby, BC Canada; 4 Centre for Global Health, Dalla Lana School of Public Health, University of Toronto, Toronto, ON Canada; 5 Department of Medicine, McMaster University, Hamilton, ON Canada; 6 Department of Pediatrics, McMaster University, Hamilton, ON Canada

**Keywords:** COVID-19, Vaccine hesitancy, South Asian, Canada, COVID-19, hésitation vaccinale, Asiatique du Sud, Canada

## Abstract

**Objective:**

South Asians represent the largest non-white ethnic group in Canada and were disproportionately impacted by the COVID-19 pandemic. We sought to determine the factors associated with vaccine hesitancy in South Asian Canadians.

**Methods:**

We conducted a cross-sectional analysis of vaccine hesitancy using data collected at the baseline assessment of a prospective cohort study, COVID CommUNITY South Asian. Participants (18 + years) were recruited from the Greater Toronto and Hamilton Area in Ontario (ON) and the Greater Vancouver Area in British Columbia (BC) between April and November 2021. Demographic characteristics and vaccine attitudes measured by the Vaccine Attitudes Examination (VAX) scale were collected. Each item is scored on a 6-point Likert scale, and higher scores reflect greater hesitancy. A multivariable linear mixed effects model was used to identify sociodemographic factors associated with vaccine hesitancy, adjusting for multiple covariates.

**Results:**

A total of 1496 self-identified South Asians (52% female) were analyzed (mean age = 38.5 years; standard deviation (SD): 15.3). The mean VAX score was 3.2, SD: 0.8 [range: 1.0‒6.0]. Factors associated with vaccine hesitancy included: time since immigration (p = 0.04), previous COVID-19 infection (p < 0.001), marital status (p < 0.001), living in a multigenerational household (p = 0.03), age (p = 0.02), education (p < 0.001), and employment status (p = 0.001).

**Conclusion:**

Among South Asians living in ON and BC, time since immigration, prior COVID-19 infection, marital status, living in a multigenerational household, age, education, and employment status were associated with vaccine hesitancy. This information can be used to address vaccine hesitancy in the South Asian population in future COVID-19 waves or pandemics.

**Supplementary Information:**

The online version contains supplementary material available at 10.17269/s41997-024-00885-7.

## Résumé

**Objectif:**

Les Asiatiques du Sud, qui représentent le plus grand groupe ethnique non-blanc au Canada, ont été démesurément touchés par la pandémie de COVID-19. Nous avons cherché à déterminer les facteurs associés à l’hésitation vaccinale chez les Canadiennes et les Canadiens asiatiques du Sud.

**Méthode:**

Nous avons mené une analyse transversale de l’hésitation vaccinale à l’aide des données collectées durant l’évaluation préliminaire d’une étude de cohorte prospective du nom de COVID CommUNITY South Asian. Les personnes participantes (18 ans et plus) ont été recrutées dans la région du grand Toronto et de Hamilton, en Ontario, et dans la région du Grand Vancouver, en Colombie-Britannique, entre avril et novembre 2021. Le profil démographique et les attitudes face aux vaccins, mesurées selon l’échelle Vaccine Attitudes Examination (VAX), ont été obtenus. Chaque élément a été noté selon une échelle de Likert en 6 points (plus la note est élevée, plus l’hésitation vaccinale est importante). Un modèle linéaire multivarié à effets mixtes a servi à identifier les facteurs sociodémographiques associés à l’hésitation vaccinale, en rajustant les données pour tenir compte de plusieurs covariables.

**Résultats:**

En tout, 1 496 personnes s’identifiant comme étant Asiatiques du Sud (dont 52 % de femmes) ont été analysées (âge moyen = 38,5 ans; écart-type [S] : 15,3). La note VAX moyenne était de 3,2, S : 0,8 [intervalle : 1,0‒6,0]. Les facteurs associés à l’hésitation vaccinale étaient : le temps écoulé depuis l’immigration (*p* = 0,04), une infection antérieure par la COVID-19 (*p* < 0,001), l’état matrimonial (*p* < 0,001), le fait de vivre dans un ménage multigénérationnel (*p* = 0,03), l’âge (*p* = 0,02), l’instruction (*p* < 0,001) et la situation d’emploi (*p* = 0,001).

**Conclusion:**

Chez les Asiatiques du Sud vivant en Ontario et en Colombie-Britannique, le temps écoulé depuis l’immigration, une infection antérieure par la COVID-19, l’état matrimonial, le fait de vivre dans un ménage multigénérationnel, l’âge, l’instruction et la situation d’emploi étaient associés à l’hésitation vaccinale. Ces informations peuvent être utilisées pour aborder l’hésitation vaccinale dans la population asiatique du Sud lors de vagues de COVID-19 ou de pandémies futures.

## Introduction

Vaccine hesitancy is defined by the World Health Organization (WHO) as the “delay in acceptance or refusal of vaccines despite the availability of vaccine services”, and reflects a continuum of attitudes from those who accept all vaccines with confidence and those who refuse all vaccines with conviction (MacDonald, 2015). WHO identified vaccine hesitancy as one of the top ten threats to global health in 2019; its relevance is heightened by the global COVID-19 pandemic (MacDonald, 2015). Vaccine hesitancy can also be viewed as a multifactorial decision-making process that is highly intersectional and influenced by various scientific, socioeconomic, psychological, sociocultural, and political factors (Dubé et al., 2016). Due to the complex intersection of factors, vaccine hesitancy varies across different contexts, time periods, and populations (MacDonald, 2015). Reasons for vaccine hesitancy may include vaccine safety concerns, the perception that vaccines were developed too rapidly to be safe, and concern over potential long-term side effects. Vaccine hesitancy has been shown to vary by sociodemographic factors including age, gender, education, income, and ethnicity, among others (Cascini et al., 2021; Cénat et al., 2022).

In Canada, 80% of adults received at least two COVID-19 vaccine doses (Public Health Agency of Canada, 2021). Despite the high estimated vaccine coverage, vaccine hesitancy may exist among those who choose to get vaccinated, illustrating the complexities of vaccine hesitancy (Reece et al., 2023). Existing literature on COVID-19 vaccine hesitancy in Canada is consistent with that of general vaccines. One consistent finding was that vaccine hesitancy was higher among racialized populations (Cénat et al., 2022). However, few studies in Canada present disaggregated results by racial or ethnic group, as these characteristics are not collected routinely in healthcare administrative datasets, or as a result of limitations in sample size, therefore preventing a nuanced understanding of vaccine hesitancy among ethnically diverse communities in Canada.

There is little literature on COVID-19 vaccine hesitancy in individual ethnic groups in Canada, including the South Asian community. South Asians are people who originate from the Indian subcontinent. They are the largest non-white ethnic group in Canada, representing more than one third of visible minorities in Ontario, and more than one quarter of visible minorities in British Columbia (BC) (Statistics Canada, 2017). South Asians were overrepresented among COVID-19 cases in Ontario, accounting for over 16% of COVID-19 cases from 26 June 2020 to 21 April 2021, yet made up only 8.6% of the population (McKenzie et al., 2021). In the Canadian Community Health Survey (June 2021 through February 2022), 96% of South Asians received at least 1 dose of the COVID-19 vaccine compared to 93% of those who were not visible minorities (Public Health Agency of Canada, 2021). This likely reflects the outreach work done across Canada during this unprecedented vaccine rollout (Song et al., 2023).

There are few reports of vaccine hesitancy among South Asians in Canada and those who choose to get vaccinated (Ochieng et al., 2021; Reece et al., 2023). Understanding the factors associated with COVID-19 vaccine hesitancy is a necessary step towards the development of tailored strategies to improve vaccine uptake for future doses of the COVID-19 vaccines in this high-risk group, and for future waves of COVID-19 or other pandemics.

This study aimed to identify factors associated with vaccine hesitancy in the South Asian community in the Greater Toronto Hamilton Area (GTHA) and Greater Vancouver Area (GVA) in Canada.

## Methods

A cross-sectional analysis of COVID CommUNITY South Asian, a prospective cohort study of South Asian adults in the GTHA in Ontario and GVA in BC was performed. The purpose of the study was to investigate vaccine access, immunogenicity, effectiveness, safety, and hesitancy among high-risk populations, including South Asians. The study was approved by the Hamilton Integrated Research Ethics Board (13323 — March 24, 2021) and the British Columbia Research Ethics Board (H21-00866—June 18, 2021).

### Participants

**Fig. 1:**
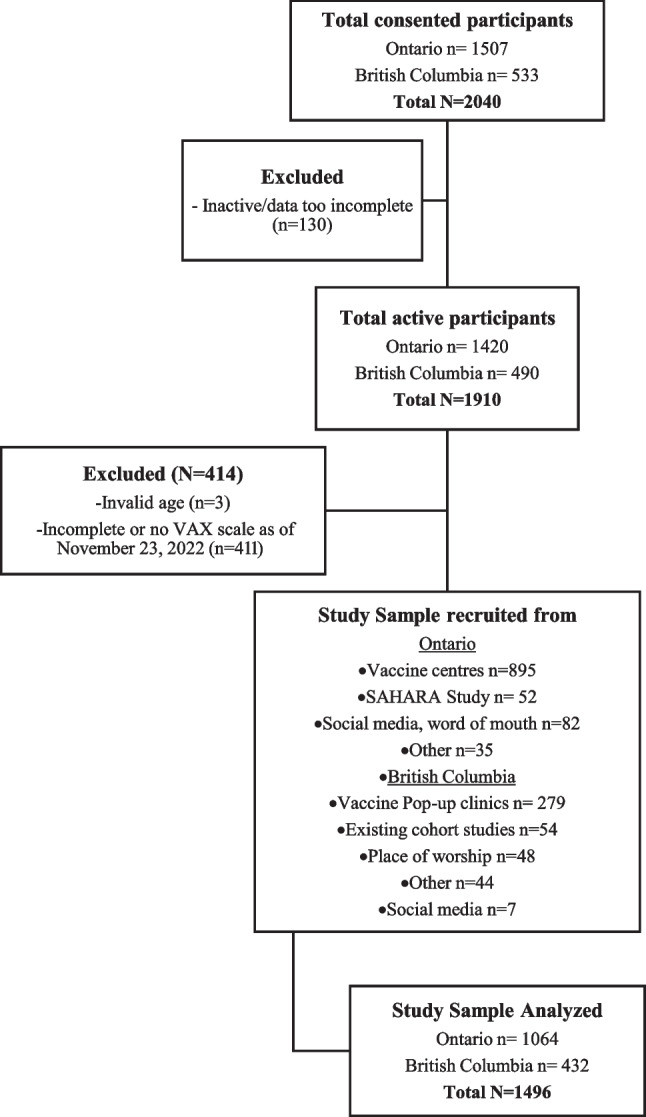
CONSORT diagram

Adults (18 + years) of self-identified South Asian ethnicity and having provided informed consent were eligible for the study (see screening form, available in ESM-1). Self-reported South Asian ethnicity was defined by parental South Asian ancestry from the Indian subcontinent, Africa, Caribbean, and/or Guyana. Recruitment primarily occurred from vaccine centres after receiving the vaccine, with a smaller proportion from places of worship, word of mouth/social media, and invitation of South Asian participants from existing cohort studies. In Ontario, recruitment spanned from 14 April to 30 October 2021, and in BC, from 21 June to 23 November 2021. Participants who completed the Vaccine Attitudes Examination (VAX) scale within 12 months of recruitment were included in this analysis (Fig. 1).

### Setting

In Ontario and BC, COVID-19 vaccine rollout occurred in a phased approach beginning in December 2020, prioritizing high-risk populations (e.g., long-term care residents, healthcare workers). Vaccines were available to the general adult population from May 2021 onwards, with COVID-19 vaccination mandates implemented by the federal government for international travel in October 2021, by both provincial governments for entering public settings or workplaces, and by individual employers, requiring mandatory vaccination starting from September 2021.

### Data collection

At enrolment, research personnel collected sociodemographic information and vaccination status. Participants were encouraged to complete an in-person or online questionnaire (see ESM-2) via an emailed link. The questionnaire collected additional information regarding employment type, health history, prior SARS-CoV-2 infection, and general vaccine attitudes. If participants did not complete the questionnaire online, study staff contacted participants, asking them to complete the questionnaire and administering a subset of questions by telephone (first visit short questionnaire, available in ESM-2). Although the questionnaires were provided in English, research personnel, many of whom were of South Asian origin and spoke multiple South Asian languages, provided translation in participants’ preferred language upon request.

### Measures

Sociodemographic factors collected included age, sex at birth, highest level of education completed, employment status, marital status, time since immigration, multigenerational household status, previous self-reported COVID-19 infection, past medical history, and median neighbourhood household income.

Attitudes towards vaccines were measured using the VAX scale (Martin & Petrie, 2017). The VAX scale was developed to assess attitudes towards vaccines in general, and comprises 12 items categorized into 4 subscales: mistrust of vaccine benefit, worries about unforeseen future side effects, concerns about commercial profiteering, and preference for natural immunity (Martin & Petrie, 2017). These items were scored on a 6-point Likert scale with 1 indicating strongly disagree and 6 indicating strongly agree. Higher scores were interpreted as more vaccine hesitant. The VAX scale has been validated in Italian, French, Spanish, Romanian, and Turkish populations within the COVID-19 context (Bruno et al., 2022; Eisenblaetter et al., 2023; Espejo et al., 2022; Huza, 2020; Yildiz et al., 2021) and has reported good internal consistency and sufficient convergent validity, and construct validity (Martin & Petrie, 2017).

### Statistical analysis

Standard descriptive statistics were calculated for sociodemographic variables and VAX scores. Cronbach’s alpha values were calculated to assess the internal consistency of the VAX subscales with the overall scale. Confirmatory factor analysis (CFA) using SPSS Amos (v26) was performed to evaluate the construct validity of the VAX scale. Model fit was evaluated using the Comparative Fit Index (CFI) (≥ 0.95), Tucker-Lewis Index (TLI) (≥ 0.95), and Root Mean Square Error of Approximation (RMSEA) (≤ 0.006) (Hu & Bentler, 1999).

Non-responders to the VAX scale were compared with responders by age, sex, education, and income. Those who selected “prefer not to answer” for the time since immigration, employment, and marital status were compared to those who provided responses with respect to age, sex, education, and income.

A multivariable linear mixed effects model was constructed to identify sociodemographic factors associated with vaccine hesitancy and included a random effect for household to account for the correlation among multiple respondents within the same household. Variables were included in the multivariable models if the univariable relationship between sociodemographic factors and the mean VAX score had a p-value ≤ 0.20. Listwise deletion was used to handle missing data for variables missing less than 5% data. Where 5% or more of data were missing for a question, i.e., for time since immigration (13%), we included a “missing indicator” category. In the multivariable linear mixed effects model, variables with a p value of < 0.05 were considered statistically significant. Statistical analysis was conducted using SPSS v28 (2021).

## Results

Between April and November 2021, a total of 2040 participants were enrolled into the COVID CommUNITY study, of whom 1910 participants were active and had complete data, and 1496 participants (73%) provided complete responses to the VAX scale and were included in the analysis (Fig. 1). There were 1339 unique households with 1194 households having a single respondent. 71.1% (n = 1064) of participants were from Ontario and 28.9% (n = 432) of participants were from BC. Participants were recruited from vaccine centres (78%), places of worship (3%), social media and word of mouth (6%), and existing cohort studies (7%). Participants who did not complete the VAX scale were more likely to be male, be from Ontario, and have higher income levels. No important differences in age and education were observed (Supplementary Table 1).

The Cronbach’s α for the 12-item VAX scale was 0.85, indicating good internal consistency. The Cronbach’s alpha across the four subscales ranged from 0.78 to 0.90 (Supplementary Table 2). The CFA demonstrated that the model was a good fit [CFI = 0.97; TLI = 0.96; RMSEA = 0.06, 90% CI = 0.05, 0.06] (Supplementary Fig. 1).

### Demographic characteristics

The mean age of participants was 38.5 ± 15.3 years and 52.2% were female (Table 1). The most common mother tongue languages reported were Punjabi (49.3%), Hindi (10.9%), and Urdu (9.8%). The study participants were highly educated with over half of participants (59.1%) reporting having a university degree. Approximately two thirds of participants (64.6%) were employed, while 68.4% had a median neighbourhood household income of over $80,000. About half of participants (51.9%) were born in Canada or had lived in Canada for more than 10 years. Most participants reported not living in a multigenerational household (74.5%) and having no previous COVID-19 infection (82.3%). Nearly all (99.2%) participants received at least one dose of the COVID-19 vaccine.

**Table 1:** Demographic characteristics of study participants (N = 1496)

Demographic factors	N (%)
Age	
18‒24	308 (20.6%)
25‒34	413 (27.6%)
35‒44	309 (20.7%)
45‒54	211 (14.1%)
55‒64	134 (9.0%)
65 +	117 (7.8%)
Missing	4 (0.3%)
Sex	
Male	701 (46.9%)
Female	781 (52.2%)
Prefer to self-describe	3 (0.2%)
Prefer not to answer	8 (0.5%)
Missing	3 (0.2%)
Mother tongue	
Punjabi	738 (49.3%)
Hindi	163 (10.9%)
Urdu	147 (9.8%)
English	140 (9.4%)
Gujarati	130 (8.7%)
Tamil	77 (5.1%)
Bengali	29 (1.9%)
Other South Asian languages	118 (7.9%)
Prefer not to answer	8 (0.5%)
Highest level of education completed	
Less than high school graduation	32 (2.1%)
High school graduate	281 (18.8%)
Trade certificate, vocational school, or apprenticeship training	32 (2.1%)
Non-university certificate or diploma from a community college, CEGEP	180 (12.0%)
University bachelor’s degree	515 (34.4%)
University graduate degree (e.g., masters or doctorate)	370 (24.7%)
Prefer not to answer	78 (5.2%)
Missing	8 (0.5%)
Marital status	
Never married	448 (29.9%)
Common law/Living with partner	39 (2.6%)
Currently married	856 (57.2%)
Divorced/Separated	47 (3.1%)
Widowed	23 (1.5%)
Prefer not to answer	75 (5.0%)
Missing	8 (0.5%)
Employment status	
Employed	967 (64.6%)
Unemployed	200 (13.4%)
Retired	98 (6.6%)
Temporarily laid off due to COVID-19	29 (1.9%)
Permanently laid off due to COVID-19	9 (0.6%)
Prefer not to answer	185 (12.4%)
Missing	8 (0.5%)
Median neighbourhood household income	
$0‒$39,999	0
$40,000‒$59,999	49 (3.3%)
$60,000‒$79,999	415 (27.7%)
$80,000‒$99,999	775 (51.8%)
$100,000 +	248 (16.6%)
Missing	9 (0.6%)
Time since immigration	
Born in Canada	199 (13.3%)
10 years or more	578 (38.6%)
5–10 years	147 (9.8%)
< 5 years	324 (21.7%)
Prefer not to answer	58 (3.9%)
Missing	190 (12.7%)
Multigenerational household	
Yes	202 (13.5%)
No	1114 (74.5%)
Prefer not to answer	166 (11.1%)
Missing	14 (0.9%)
Any recorded infection of COVID-19	
Yes	246 (16.4%)
No	1231 (82.3%)
Prefer not to answer	15 (1.0%)
Missing	4 (0.3%)
Medical history of cardiovascular disease (myocardial infarction, angioplasty, or stroke)	
Yes	40 (2.7%)
No	1449 (96.9%)
Missing	7 (0.5%)
Chronic medical conditions requiring medication	
Diabetes	
Yes	93 (6.2%)
No	1376 (92.0%)
Missing	27 (1.8%)
Hypertension	
Yes	102 (6.8%)
No	1367 (91.4%)
Missing	27 (1.8%)
Heart disease or stroke	
Yes	19 (1.3%)
No	1450 (96.9%)
Missing	27 (1.8%)
Arthritis	
Yes	22 (1.5%)
No	1447 (96.7%)
Missing	27 (1.8%)
Chronic lung disease	
Yes	3 (0.2%)
No	1466 (98.0%)
Missing	27 (1.8%)
Vaccinated against COVID-19	
No	9 (0.6%)
Yes, one or more doses*	1486 (99.2%)
Prefer not to answer	1 (0.1%)
Missing	4 (0.3%)

^*^78% of participants were recruited at vaccine centres

The mean overall VAX score was 3.2 ± 0.8 points [range: 1.0–6.0] (Table 2). Greater vaccine hesitancy is indicated by higher VAX scores. The highest mean VAX score was in the “worries about unforeseen future effects” subscale with a score of 4.1 ± 1.1 points, followed by the “preference for natural immunity” subscale with a score of 3.5 ± 1.3 points. Lower scores reflecting less hesitancy were observed in the “concerns about commercial profiteering” subscale (2.9 ± 1.2 points), with the least hesitancy regarding the “mistrust of vaccine benefit” subscale (2.2 ± 1.1 points). No important differences in VAX scores between South Asians living in Ontario and those living in BC were observed, except for the “mistrust of vaccine benefit” subscale, which reflected greater mistrust among BC participants.

**Table 2:** VAX scale characteristics of participants (N = 1496)

	Mean VAX score (SD)		
	Overall (N = 1496)	Ontario (n = 1064)	British Columbia (n = 432)
Overall VAX score	3.2 (0.8)	3.2 (0.8)	3.2 (0.9)
Mistrust of vaccine benefit subscale	2.2 (1.1)	2.1 (1.0)	2.4 (1.2)
Worries about unforeseen future effects subscale	4.1 (1.1)	4.1 (1.1)	4.1 (1.1)
Concerns about commercial profiteering subscale	2.9 (1.2)	2.9 (1.2)	3.0 (1.3)
Preference for natural immunity subscale	3.5 (1.3)	3.5 (1.3)	3.5 (1.3)

VAX: Vaccine Attitudes Examination

### Multivariable analysis

**Fig. 2:**
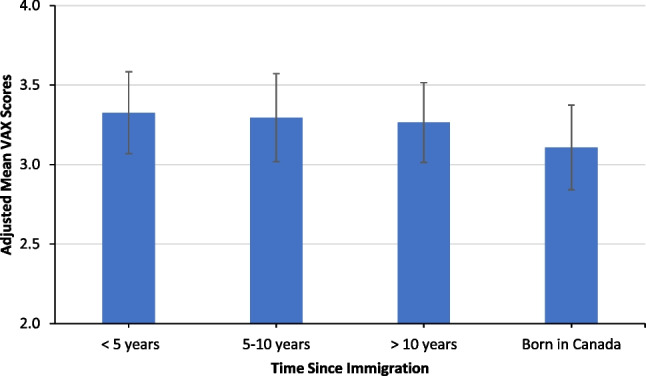
Adjusted mean VAX scores and 95% confidence intervals by time since immigration

The associations of sociodemographic factors with vaccine hesitancy measured by the VAX scale and adjusted mean VAX scores of sociodemographic factors are shown in Table 3 and Fig. 2. Time since immigration (p = 0.04), previous COVID-19 infection (p < 0.001), marital status (p < 0.001), living in a multigenerational household (p = 0.03), age (p = 0.02), education (p < 0.001), and employment status (p = 0.001) were each associated with VAX scores (Table 3).

**Table 3:** Multivariable linear mixed effects model of sociodemographic factors associated with vaccine hesitancy measured by VAX Score

Factor	Parameter estimate	95% confidence interval	P-value
Time since immigration			0.04
Born in Canada (Reference group)			
> 10 years in Canada	0.16	(0.01, 0.30)	
5‒10 years in Canada	0.19	(0.01, 0.37)	
< 5 years in Canada	0.22	(0.07, 0.37)	
Prefer not to answer	0.33	(0.08, 0.58)	
Missing	0.20	(0.03, 0.37)	
Previous COVID-19 infection			< 0.001
No (Reference group)			
Yes	0.22	(0.11, 0.34)	
Prefer not to answer	0.03	(-0.38, 0.43)	
Marital status			< 0.001
Never married (Reference group)			
Currently married/Common law/Living with partner	0.28	(0.16, 0.40)	
Previously married	0.50	(0.28,0.73)	
Prefer not to answer	0.44	(0.21, 0.68)	
Multigenerational household			0.03
No (Reference group)			
Yes	-0.11	(-0.23, 0.02)	
Prefer not to answer	0.13	(-0.01, 0.27)	
Sex at birth			0.40
Male (Reference group)			
Female	-0.04	(-0.12, 0.04)	
Prefer not to answer	-0.30	(-0.87, 0.27)	
Age (per 10 years)	-0.05	(-0.1, -0.01)	0.02
Highest level of education completed			< 0.001
High school graduate or less (Reference group)			
Non-academic or vocational education and training	0.16	(0.02, 0.31)	
University bachelor’s or graduate degree	-0.13	(-0.23, -0.02)	
Prefer not to answer	0.02	(0.21, 0.25)	
Employment status			0.001
Unemployed (Reference group)			
Retired	-0.21	(-0.43, 0.01)	
Employed	0.07	(-0.04, 0.19)	
Prefer not to answer	0.23	(0.06, 0.40)	

Intraclass correlation coefficient of 0.45

Overall, immigrants to Canada were more hesitant compared to those born in Canada, with lower vaccine hesitancy associated with longer duration of life in Canada (Fig. 2). Compared to those born in Canada, immigrants who lived in Canada < 5 years, 5–10 years, or > 10 years scored progressively lower vaccine hesitancy points (0.22, 0.19, and 0.16, respectively). Participants reporting previous COVID-19 infection scored 0.2 points higher (95% CI: 0.1, 0.3) than those who did not report previous COVID-19 infection. Married or common-law participants scored 0.3 points higher (95% CI: 0.2, 0.4) and previously married (divorced/separated/widowed) participants scored 0.5 points higher (95% CI: 0.3, 0.7) compared to those who were never married. Participants living in a multigenerational household scored 0.1 points lower (95% CI: -0.2, 0.02) than those who reported not living in a multigenerational household. For every 10-year increase in age, there was a 0.05-point decrease (-0.1, -0.01) in the mean VAX score. For education, there was a lower vaccine hesitancy with a higher level of education completed. Those with a university degree scored 0.1 points lower (95% CI: -0.2, -0.02) and those with non-academic or vocational education and training scored 0.2 points higher (95% CI: 0.02, 0.3) than those who were high school graduates or less. Participants who were employed scored 0.07 points higher (95% CI: -0.04, 0.2) and participants who were retired scored 0.2 points lower (95% CI: -0.4, 0.01) than those who were unemployed.

Participants who selected “prefer not to answer” for time since immigration, employment status, and marital status scored higher on the VAX scale compared to those born in Canada, those unemployed, and those never married (p = 0.01, p = 0.007, and p < 0.001, respectively). Those selecting “prefer not to answer” at least once across these variables were younger, with no important differences observed in sex, income, and education (Supplementary Table 3).

Recruitment spanned seven months across both provinces, during which risk perceptions may have shifted with increased vaccine uptake due to vaccine mandates. In a sensitivity analysis, we examined differences in VAX scores before (14 April‒31 August 2021) and after (1 September‒23 November 2021) vaccine mandates. We observed that time period was associated with increased vaccine hesitancy in the second time period compared to the first, while other estimates showed minimal changes (Supplementary Table 4).

## Discussion

Among South Asians from Ontario and BC, we identified that time since immigration, marital status, prior COVID-19 infection, age, multigenerational household status, education, and employment status were independently associated with vaccine hesitancy.

We observed that newer immigrants are more hesitant than settled immigrants or South Asians born in Canada, which may reflect lower familiarity with the healthcare system and less trust of public health advice (Kalich et al., 2016). This aligns with data indicating that immigrants face barriers accessing healthcare and health information due to economic and language barriers and cultural differences, which may contribute to greater hesitancy (Kalich et al., 2016). This is consistent with Canadian studies, which also demonstrated that immigrants were more likely to be hesitant (Lin, 2022; Muhajarine et al., 2021).

We observed that married/common-law individuals were more vaccine hesitant. This may reflect vaccine safety concerns at the time, including possible future effects on fertility, pregnancy, and breastfeeding (Hsu et al., 2022). Previous COVID-19 vaccine hesitancy studies found varying results regarding the effect of marital status on vaccine hesitancy. In a global scoping review, some studies demonstrated that unmarried individuals were more hesitant, and that being married was associated with higher vaccine acceptance (Joshi et al., 2021). Our findings are consistent with other studies conducted in Canada and the United States, which demonstrate that married individuals were more vaccine hesitant (Reece et al., 2023; Santavicca et al., 2023). Further, previously married individuals may have been more hesitant due to a lack of social support.

Individuals with prior COVID-19 infection were more vaccine hesitant than those who did not report prior infection, which may be due to their preference for infection-induced immunity, colloquially referred to as “natural” immunity. This includes beliefs that natural immunity may be better than vaccination and provide greater protection, which is consistent with other COVID-19 vaccine hesitancy studies in South Asian countries (Ennab et al., 2022). Alternatively, those with prior COVID-19 infection may have also been more hesitant due to breakthrough infections and vaccine effectiveness concerns.

Younger individuals were more vaccine hesitant than older individuals, reflecting their perceptions of being healthier and feeling greater confidence of being able to “fight off the infection”. Older individuals may be less hesitant due to their increased vulnerability to COVID-19 and consequences as a result of chronic illnesses or other conditions. This may be attributed to public perceptions of risk, given that more severe and mortal outcomes during the pandemic were experienced by older individuals. A systematic review and meta-analysis found that Canadian studies demonstrated varying effects of age on COVID-19 vaccine hesitancy. Most studies, consistent with our findings, have demonstrated that vaccine hesitancy among younger age groups was likely due to lower COVID-19 risk perception (Cénat et al., 2022).

We observed that individuals living in a multigenerational household were less vaccine hesitant than those who reported not living in a multigenerational household. Although individual perspectives within a household vary, less hesitancy may be attributed to increased social support, an increased awareness of health risks affecting high-risk individuals in the family, and perceptions being informed by the community due to the collectivist nature of South Asian cultures (Akbar et al., 2022; Zhang et al., 2022),

Individuals with a university degree were observed to be less vaccine hesitant and individuals with a non-academic or vocational education and training were observed to be more hesitant compared to high school graduates or less. This non-linear relationship has also been observed in other studies (Willis et al., 2023). Individuals may be more or less hesitant across different education levels because of the risks and side effects of the vaccine, lack of knowledge, or lack of trust in the vaccine (McElfish et al., 2021).

We also observed that employed individuals were more vaccine hesitant, while retired individuals were less hesitant, compared to individuals who were unemployed. Employed individuals may be more hesitant due to concerns about potential side effects of the vaccine interfering with their ability to work (Xie et al., 2022). Retired individuals’ lower vaccine hesitancy may be in part due to older age and their increased vulnerability to COVID-19, as previously mentioned.

Previous research in Canada indicates higher COVID-19 vaccine hesitancy among those who are younger, female, married, immigrants, or racialized, or have a lower educational level (Cénat et al., 2022). Reasons for COVID-19 vaccine hesitancy among racialized people may include institutional mistrust, vaccine development concerns, lack of reliable information, and vaccine safety and effectiveness concerns (Ochieng et al., 2021). This is consistent with other research conducted in the South Asian context. A study conducted among South Asians in Ontario that aimed to identify areas for improvement in public health communication found that the lack of accessible and culturally relevant information resulted in the spread of misinformation and contributed to vaccine hesitancy (Bhalla et al., 2022). This study reveals the complexities of vaccine hesitancy, with sociodemographic factors having varying effects across different populations. This highlights the importance of disaggregated data by ethnicity and by those who choose to be vaccinated, as hesitancy may persist, particularly with booster doses. A nuanced understanding of vaccine hesitancy and a collaborative approach involving healthcare professionals, policymakers, and community leaders are essential to address the complex intersection of factors driving hesitancy. One such model emerged during the COVID-19 pandemic in Peel Region in Ontario, where the local public health unit partnered with a South Asian Taskforce that assisted with both communications and outreach as well as delivery of vaccinations in a specialized, culturally congruent clinic targeted at the South Asian population (Polsinelli, 2021).

### Strengths and limitations of the study

The strengths of our study included its large size, inclusion of South Asians in Canada from Ontario and BC and particularly in “hot-spot” regions in those two provinces, and our direct measure of ethnicity, as all study participants self-identified as South Asian. The limitations of our analysis include the non-random study sample, given that over 78% of participants were recruited from vaccine centres and > 99% were vaccinated with at least one dose of the COVID-19 vaccine, limiting the generalizability of our results to non-South Asian groups. Our estimates of vaccine hesitancy are likely an underestimate, as those who refused or delayed the vaccine at the time and those who were not as likely to present to a vaccine centre were not included. Therefore, our findings may not be representative of the entire South Asian population in Canada. However, COVID-19 vaccine mandates implemented in Ontario and BC during our study recruitment period may have captured highly hesitant individuals getting vaccinated due to vaccine mandates as shown in our sensitivity analysis. It showed that overall vaccine hesitancy in the second time period compared to the first increased, which is correlated with the timing of vaccine mandates starting in September 2021 (Supplementary Table 4). The VAX scale was designed to assess general vaccine attitudes; however, the questionnaire was administered during the COVID-19 pandemic, and participant responses may be reflective of their attitudes toward COVID-19 vaccines rather than toward vaccines in general. While it may be considered a limitation that participants who selected “prefer not to answer” were included in this analysis, we analyzed the characteristics of this group. The significant association of “prefer not to answer” and higher VAX scores highlights the influence of non-response in our findings as such participants may have chosen not to provide certain demographic information due to the sensitivity of the topic, stigma, or complexities of their immigrant status. Participants who chose “prefer not to answer” for time since immigration, employment status, and marital status had higher VAX scores and were younger but did not differ in other characteristics from responders. While our study provides insights into the South Asian population from two regions in the country, the findings have limited generalizability to other ethnic populations in Canada. Further, causality cannot be established due to the cross-sectional nature of this analysis.

## Conclusion

Among South Asians from two regions in Canada (the provinces of Ontario and British Columbia), time since immigration, marital status, prior COVID-19 infection, age, multigenerational household status, education, and employment status were each independently associated with vaccine hesitancy. This information highlights the importance of understanding the factors associated with vaccine hesitancy in non-white ethnic groups. A multi-faceted, tailored approach, including targeted messaging, building trust, and actively collaborating with community organizations with a focus on new immigrants, younger people, and other key sociodemographic factors is required to effectively address vaccine hesitancy and enhance vaccine uptake in the future.

## Contributions to knowledge

What does this study add to existing knowledge?

- This study provides insights into the sociodemographic factors associated with vaccine hesitancy among a sample of South Asians and vaccinated individuals in Canada, contributing to a limited knowledge base on vaccine hesitancy in this ethnic group.
- Results from our study demonstrate that factors associated with hesitancy include time since immigration, marital status, prior COVID-19 infection, age, multigenerational household status, education, and employment status.

What are the key implications for public health interventions, practice, or policy?

- This study conducted in a high-risk community offers valuable insights into the complexities of vaccine hesitancy. The study underscores the importance of developing and implementing tailored public health communication strategies in collaboration with communities to address unique sociodemographic factors, including a focus on new immigrants, younger people, those who are married, and those who live in multigenerational households.
- Such communication and health promotion strategies may be useful to promote vaccine uptake in the South Asian population for future COVID-19 waves or pandemics.

## Supplementary Information

Below is the link to the electronic supplementary material.

Supplementary file1 (DOCX 264 KB)

Supplementary file2 (PDF 69.4 KB)

Supplementary file3 (PDF 187 KB)

## Data Availability

The data from this study contain identifiable information that cannot be given to an outside group and are not publicly available as per the study consent guidelines. Nevertheless, requests for collaboration will be considered.
