# Supplementary material for: Sociodemographic factors associated with vaccine hesitancy in the South Asian community in Canada
Source: Can J Public Health. 2024 May 7;115(6):924–35. doi: 10.17269/s41997-024-00885-7 (PMC11638425; doi:10.17269/s41997-024-00885-7)
Supplement: Supplementary file 1 — Supplementary file1 (DOCX 264 KB) [file 41997_2024_885_MOESM1_ESM.docx]

**Online Supplementary Material**

Supplementary Table 1: Demographics in responders versus non-responders for the VAX scale

Supplementary Table 2: Alpha reliabilities of subscales and overall mean VAX score

Supplementary Table 3: Demographics in responders versus at least one “prefer not to answer” response for time since immigration, marital status, and employment status

Supplementary Table 4: Sensitivity analysis of sociodemographic factors associated with vaccine hesitancy measured by VAX score across different time periods based on VAX scale completion date

Supplementary Figure 1: Confirmatory factor analysis of VAX scale

**Supplementary Table 1:** Demographics in responders versus non-responders for the VAX scale

|  |  | **Completed VAX Scale** | |
| --- | --- | --- | --- |
|  | **Overall** | **Yes** | **No** |
| **N** | 1910 | 1496 | 414 |
| Female (%) | 51.0 | 52.7 | 46.5 |
| Age (SD) | 38.6 (15.3) | 38.5 (15.3) | 39.1 (15.3) |
| Median neighbourhood household income >=$80,000 (%) | 70.5 | 68.7 | 80.1 |
| University degree (%) | 62.9 | 63.5 | 55.6 |

**Supplementary Table 2:** Alpha reliabilities of subscales and overall mean VAX score

|  | **Cronbach’s Alpha** |
| --- | --- |
| Mistrust of vaccine benefit subscale | 0.90 |
| Worries about unforeseen future effects subscale | 0.78 |
| Concerns about commercial profiteering | 0.81 |
| Preference for natural immunity | 0.85 |
| **Overall VAX score** | **0.85** |

**Supplementary Table 3:** Demographics in responders versus at least one “prefer not to answer” response for time since immigration, marital status, and employment status

|  |  | **At least 1 “prefer not to answer” response** | |
| --- | --- | --- | --- |
|  | **Overall** | **No** | **Yes** |
| **N** | 1496 | 1257 | 239 |
| Female (%) | 52.7 | 53.1 | 50.4 |
| Age (SD) | 38.5 (15.3) | 38.8 (15.5) | 36.7 (14.0) |
| Median neighbourhood household income >=$80,000 (%) | 68.8 | 68.9 | 68.4 |
| University degree (%) | 62.8 | 62.9 | 62.1 |

Note: Those who selected “prefer not to answer” at least once across the time since immigration, marital status, and employment status questions were slightly younger, while slightly more females provided responses.

**Supplementary Table 4:** Sensitivity analysis of sociodemographic factors associated with vaccine hesitancy measured by VAX score across different time periods based on VAX scale completion date

| **Factor** | **Parameter**  **Estimate** | | | **95% Confidence Interval** | **P-Value** |  |
| --- | --- | --- | --- | --- | --- | --- |
| **Time since immigration** |  | | |  | 0.10 | |
| Born in Canada (Reference group) |  | | |  |  |  |
| >10 years in Canada | 0.16 | | | (0.01, 0.30) |  |  |
| 5-10 years in Canada | 0.19 | | | (0.01, 0.37) |  |  |
| <5 years in Canada | 0.19 | | | (0.04, 0.33) |  |  |
| Prefer not to answer | 0.31 | | | (0.06, 0.56) |  |  |
| Missing | 0.14 | | | (-0.03, 0.31) |  |  |
| **Previous COVID-19 infection** |  | | |  | 0.002 | |
| No (Reference group) |  | | |  |  |  |
| Yes | 0.20 | | | (0.09, 0.32) |  |  |
| Prefer not to answer | 0.02 | | | (-0.38, 0.42) |  |  |
| **Marital Status** |  | | |  | <0.001 | |
| Never married (Reference group) |  | | |  |  |  |
| Currently married/Common law/Living with partner | 0.27 | | | (0.15, 0.39) |  |  |
| Previously married | 0.49 | | | (0.26,0.71) |  |  |
| Prefer not to answer | 0.42 | | | (0.19, 0.65) |  |  |
| **Multigenerational household** |  | | |  | 0.05 | |
| No (Reference group) |  | | |  |  |  |
| Yes | -0.10 | | | (-0.23, 0.02) |  |  |
| Prefer not to answer | 0.12 | | | (-0.02, 0.25) |  |  |
| **Sex at birth** |  | | |  | 0.35 | |
| Male (Reference group) |  | | |  |  |  |
| Female | -0.04 | | | (-0.12, 0.04) |  |  |
| Prefer not to answer | -0.33 | | | (-0.89, 0.24) |  |  |
| **Age (per 10 years)** | -0.04 | | | (-0.09, 0.001) | 0.06 |  |
| **Highest Level of Education Completed** |  | | |  | <0.001 | |
| High school graduate or less (Reference group) |  | | |  |  |  |
| Non-academic or vocational education and training | 0.17 | | | (0.03, 0.31) |  |  |
| University bachelor’s or graduate degree | -0.11 | | | (-0.22, -0.01) |  |  |
| Prefer not to answer | 0.03 | | | (0.20, 0.25) |  |  |
| **Employment Status** |  | | |  | 0.002 | |
| Unemployed (Reference group) |  | | |  |  |  |
| Retired | -0.22 | | | (-0.44, -0.001) |  |  |
| Employed | 0.06 | | | (-0.06, 0.17) |  |  |
| Prefer not to answer | 0.23 | | | (0.06, 0.40) |  |  |
| **Time period based on VAX scale completion** | | | | | <0.001 |  |
| Pre-vaccine mandates: April 14, 2021 to August 31, 2021 (Reference group) | |  |  | |  |  |
| Post-vaccine mandates: September 1, 2021 to November 23, 2021 | | 0.19 | (0.11, 0.28) | |  |  |


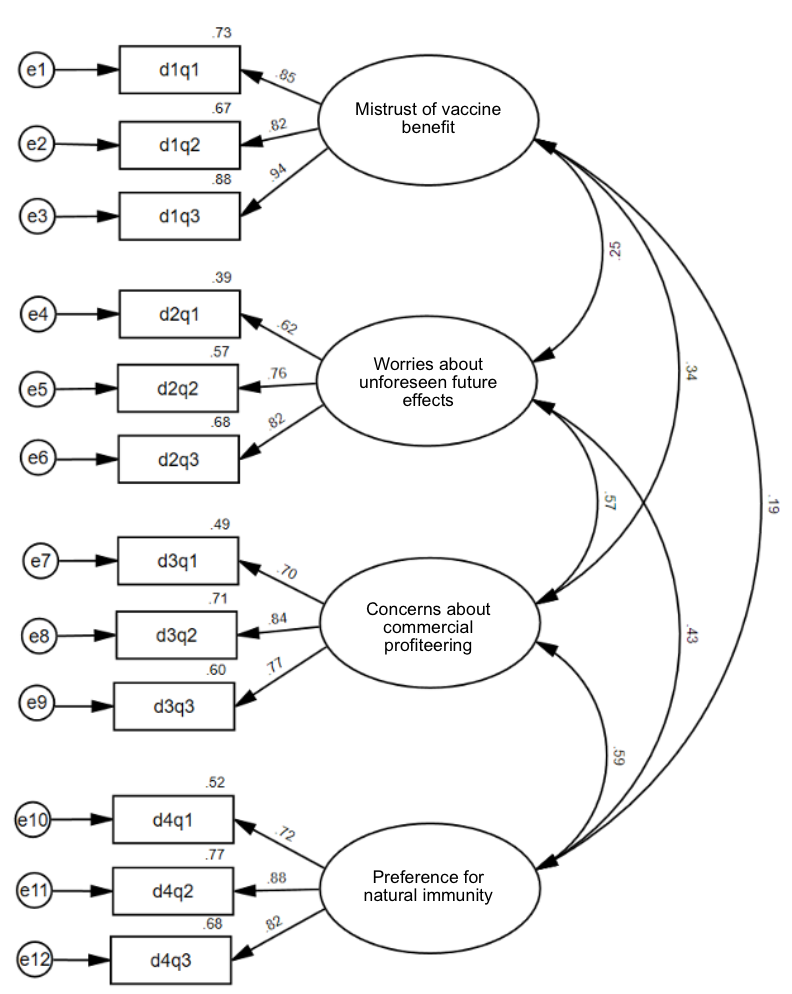


**Supplementary Figure 1:** Confirmatory factor analysis of VAX scale
