# Supplementary material for: Sociodemographic factors associated with vaccine hesitancy in the South Asian community in Canada
Source: Can J Public Health. 2024 May 7;115(6):924–35. doi: 10.17269/s41997-024-00885-7 (PMC11638425; doi:10.17269/s41997-024-00885-7)
Supplement: Supplementary file 2 — Supplementary file2 (PDF 69.4 KB) [file 41997_2024_885_MOESM2_ESM.pdf]

COVID CommUNITY - South Asian screening

Welcome and thank you for your interest in the COVID CommUNITY study.

This is an online consent form that you will need to complete to continue with the COVID CommUNITY study. Please read each section carefully.

A study team member should be with you to discuss the consent with you and answer any questions you may have.

Thank you and please do not hesitate to contact the study team if you have any questions.

CONTACT INFORMATION

TODAY	________________________
[name_text]	________________________
Name question text.	________________________
Are you at least 18 years old?	◯ Yes<br>◯ No
Do you self-identify as South Asian?	◯ Yes<br>◯ No
Email address	________________________
OPTIONAL: Email address of Witness (Translator)<br><br>If you would like to have someone you know and trust read the consent form with you to make sure you fully understand it before signing, please enter their email address here. This email address will only be used to send them the consent form to read and sign as a witness.	________________________
Where did you find out about us?	◯ Brampton soccer centre immunization clinic<br>◯ Embassy Grand Convention Centre immunization clinic<br>◯ Embassy Grand Convention Centre Testing Site<br>◯ Through another study<br>◯ Social Media<br>◯ Word of mouth (friend or family)<br>◯ Pharmacy<br>◯ Other, specify: ______
Where did you find out about us?	◯ BC pop up clinics<br>◯ Through another study<br>◯ Social Media<br>◯ Place of Worship<br>◯ Other, please specify: ______

Appendix 1, as supplied by the authors. Appendix to: Anand SS, Arnold C, Bangdiwala SI, et al. Seropositivity and risk factors for SARS-CoV-2 infection in a South Asian community in Ontario: a cross-sectional analysis of a prospective cohort study. *CMAJ Open* 2022. doi: 10.9778/cmajo.20220031. Copyright © 2022 The Author(s) or their employer(s). To receive this resource in an accessible format, please contact us at cmajgroup@cmaj.ca.

Powered by REDCap

Please specify which social media platform you were on when you heard about us:	◯ Facebook<br>◯ Instagram<br>◯ Twitter<br>◯ WhatsApp<br>◯ Other, please specify: ______
Is this your final COVID-19 vaccine dose?	◯ Yes<br>◯ No
Which study are you already a part of?	◯ SAHARA<br>◯ START<br>◯ RICH Legacy<br>◯ Canadian Alliance for Healthy Hearts and Minds (CAHHM)
Which study are you already a part of?	◯ SAHARA<br>◯ MCHAT<br>◯ RICH Legacy
DAG	__________________________________
Study ID	__________________________________
Parent Cohort Referral number	__________________________________

Sorry, you are not eligible to participate in this study.
Thank you for your interest.

Powered by REDCap
