# Supplementary material for: Sociodemographic factors associated with vaccine hesitancy in the South Asian community in Canada
Source: Can J Public Health. 2024 May 7;115(6):924–35. doi: 10.17269/s41997-024-00885-7 (PMC11638425; doi:10.17269/s41997-024-00885-7)
Supplement: Supplementary file 3 — Supplementary file3 (PDF 187 KB) [file 41997_2024_885_MOESM3_ESM.pdf]

Primary Survey

Thank you for your interest in participating in the COVID-19 Assessment. This assessment will help us understand the impact of COVID-19 in our South Asian families and community. Through these surveys, we hope to explore reasons behind the increased burden of disease in our communities and barriers around the COVID-19 vaccine. This information will help inform public health policy.

If you need to leave before you are finished these questions, please click "Save & Return Later" at the bottom of the screen. We will then send you an email link to continue with the survey.

If you have any questions about this survey, please contact our study team.

StudyID ______________________

Study email address (for participant to contact) ______________________

Date ______________________

Age ______________________

Did the participant consent to receive the InterHeart report?

◯ Yes
◯ No

Email address ______________________

DAG ______________________

---

1 What was your assigned sex at birth?

◯ Male
◯ Female
◯ Prefer to self-describe: ______
◯ Prefer not to answer

---

2 What is your sex now?

◯ Male
◯ Female
◯ Prefer to self-describe: ______
◯ Prefer not to answer

Appendix 2, as supplied by the authors. Appendix to: Anand SS, Arnold C, Bangdiwala SI, et al. Seropositivity and risk factors for SARS-CoV-2 infection in a South Asian community in Ontario: a cross-sectional analysis of a prospective cohort study. *CMAJ Open* 2022. doi: 10.9778/cmajo.20220031. Copyright © 2022 The Author(s) or their employer(s). To receive this resource in an accessible format, please contact us at cmajgroup@cmaj.ca.

1 What is your mother tongue?

☐ Bengali
☐ English
☐ Gujarati
☐ Hindi
☐ Punjabi
☐ Tamil
☐ Urdu
☐ Other, specify: ______
☐ Prefer not to answer

2 What is your religious affiliation?

☐ Buddhism
☐ Christianity
☐ Hinduism
☐ Islam
☐ Jainism
☐ Sikhism
☐ Other, specify: ______
☐ Prefer not to answer

7 Were you born in Canada?

◯ Yes
◯ No
◯ Prefer not to answer

2 Which vaccine did you receive?

◯ Pfizer
◯ Moderna
◯ Astra Zeneca
◯ Johnson & Johnson
◯ Other, specify: ______

a Date of first vaccine
If not today, you can check your vaccine card to get this date! ________________________________

b Date of second vaccine ______ ______
If you are waiting for your second dose, please enter the appointment date for your second dose.

c Which vaccine did you receive for your third dose (booster shot)?

◯ Pfizer
◯ Moderna
◯ Not applicable (no booster shot)

d Date of third vaccine, if applicable
This is intended to collect the date of your first booster vaccine, if applicable. If you have not received a booster COVID-19 shot, please leave blank. ________________________________

Have you had a positive COVID-19 test result?

◯ Yes
◯ No
◯ Prefer not to answer

---

show physical measures form

◯ True
◯ False

Powered by REDCap

Demographics Part 1 (5-10 minutes)

If you need to leave before you are finished these questions, please click "Save & Return Later" at the bottom of the screen. We will then send you an email link to continue with the survey.

If you have any questions about this survey, please contact our study team at [studyemail]

Today

____

What is your preferred language?

☐ Bengali
☐ English
☐ Gujarati
☐ Hindi
☐ Punjabi
☐ Tamil
☐ Urdu
☐ Other, specify: ______
☐ Prefer not to answer

---

3 What is the highest level of education you have completed?

◯ Less than high school graduation
◯ High school graduate
◯ Trade certificate, vocational school, or apprenticeship training
◯ Non-university certificate or diploma from a community college, CEFEP
◯ University bachelor's degree
◯ University graduate degree (e.g. masters or doctorate)
◯ Prefer not to answer

---

4 What is your marital status?

◯ Never Married
◯ Common law/Living with partner
◯ Currently married
◯ Divorced/Separated
◯ Widowed
◯ Prefer not to answer

---

5 What is your current employment status?

◯ Employed, please specify where you work: ______
◯ Unemployed
◯ Retired
◯ Temporarily laid off due to COVID-19
◯ Permanently laid off due to COVID-19
◯ Prefer not to answer

Powered by REDCap

a Have you been working in any of the following occupations or worksites in the past year?

☐ Hospital or Health Care Facility
☐ First Responder (Paramedic/Firefighter/Police Officer)
☐ Childcare Worker
☐ Correctional Officer
☐ Teacher of Other School Staff
☐ Transit Driver
☐ Transport Driver/Truck Driver
☐ Food Service Industry
☐ Grocery Store
☐ Pharmacy
☐ Hairdresser or Barber
☐ Aesthetician
☐ Flight Attendant
☐ International Airport
☐ Factory Worker
☐ Processing Centre/Shipping Warehouse
☐ Other, specify: ______
☐ Prefer not to answer

Have you been working in any of the following occupations or worksites in the past year? Other, specify: __________________________________
Site Codes:

1. Essential Worker

0. Working from home or not essential

Definitions taken from lists for the ON vaccination priority (https://covid-19.ontario.ca/ontarios-covid-19-vaccination-plan), first and second group

6 What is your best estimate of the total income of ALL household members from ALL sources in the past 12 months (before taxes)?

◯ $0 - 14, 999
◯ $15,000 - $19,000
◯ $20,000 - $29,999
◯ $30,000 - $39,000
◯ $40,000 - 49,999
◯ $50,000 - 59,999
◯ $60,000 and above
◯ Prefer not to answer

6 What is your best estimate of the total income of ALL household members from ALL sources in the past 12 months (before taxes)?

◯ $0 - 19,999
◯ $20,000 - 39,999
◯ $40,000 - 59,999
◯ $60,000 - 79,999
◯ $80,000 - 99,999
◯ $100,000 and above
◯ Prefer not to answer

a What is your country of birth?

◯ India
◯ Pakistan
◯ Sri Lanka
◯ Bangladesh
◯ Guyana
◯ Trinidad
◯ Uganda
◯ Tanzania
◯ South Africa
◯ United Arab Emirates
◯ Other, specify: ______
◯ Prefer not to answer

---

b How many years have you lived in Canada?

◯ < 5 years
◯ 5 - 10 years
◯ > 10 years
◯ Prefer not to answer

Health History (5-10 minutes)

Please continue answering the following questions.

If you need to leave before you are finished these questions, please click "Save & Return Later" at the bottom of the screen. We will then send you an email link to continue with the survey.

If you have any questions about this survey, please email our study team at covid.community.on@phri.ca

	Today	____________________
1	Have you ever...	
a	Had a heart attack?	◯ Yes ◯ No ◯ Unsure
i	When was your most recent heart attack? ______ ______ ______	
b	Had angioplasty, stent in your heart, or heart bypass surgery?	◯ Yes ◯ No ◯ Unsure
i	When was your most recent angioplasty, stent in your heart, or heart bypass surgery? ______ ______ ______	
c	Suffered a stroke?	◯ Yes ◯ No ◯ Unsure
i	When was your most recent stroke? ______ ______ ______	
d	Suffered a warning stroke (transient ischemic attack TIA)?	◯ Yes ◯ No ◯ Unsure
i	When was your most recent warning stroke? ______ ______ ______	
e	Developed heart failure?	◯ Yes ◯ No ◯ Unsure
i	When was your most recent heart failure? ______ ______ ______	
f	Had any other cardiovascular hospitalizations, e.g. angina (severe pain or discomfort), heart valve surgery, or arrhythmia?	◯ Yes ◯ No ◯ Unsure
i	When was your most recent other cardiac condition? ______ ______ ______	
	Been diagnosed with cancer?	◯ Yes ◯ No ◯ Unsure
i	When was your most recent cancer diagnosis? ______ ______ ______	
2	Do you have a chronic medical condition that requires you to take medication(s) everyday?	◯ Yes ◯ No ◯ Prefer not to answer

Powered by REDCap

a Please specify which condition(s) require you to take medication everyday.

☐ Diabetes
☐ High blood pressure
☐ Heart disease or Stroke
☐ Arthritis
☐ Chronic Lung Disease
☐ Cancer
☐ Other, specify: ______

b Please select all of the following medications that you take to manage your chronic disease(s)

☐ Ace-inhibitors to lower blood pressure (ex: benazepril, captopril, enalapril, lisinopril, ramipril)
☐ Angiotensin II Receptor Blockers to lower blood pressure (ex: canderstan, losartan, telmisartan, valsartan)
☐ Antibiotics
☐ Antivirals used for things like COVID-19 or HIV (lopinavir-ritonavir, remdesivir)
☐ Allergy medications
☐ Androgen deprivation therapy (e.g. for prostate cancer)
☐ Aspirin
☐ Asthma medications
☐ Diabetes medications
☐ Immunosuppressive or immunomodulatory medication (ex: corticosteroids, disease-modifying anti-rheumatic drugs such as adalimumab, azathioprine, ciclosporin, etanercept, infliximab, methotrexate, rituximab, sulfasalazine, tocilizumab, anti-cytokine antibodies, interferons)
☐ Non-steroidal anti-inflammatory drugs (ex: ibuprofen such as Advil or Motrin; naproxen such as Aleve)
☐ Statins (to lower cholesterol)
☐ Traditional Medicinal treatments
☐ Other, please specify: ______
☐ Don't know/Unsure
☐ Prefer not to answer

Are you currently pregnant? ◯ Yes ◯ No

How many gestational weeks pregnant are you?

____

Powered by REDCap

Vaccine Specific Questions (5-10 minutes)

If you need to leave before you are finished these questions, please click "Save & Return Later" at the bottom of the screen. We will then send you an email link to continue with the survey.

If you have any questions about this survey, please email our study team at covid.community.on@phri.ca

TODAY ________________________________

For the following questions, please choose a number that corresponds to how much you agree or disagree with the following statements, with 1 being "strongly agree" and 6 being "strongly disagree"

Domain 1: Mistrust of vaccine benefit

	Strongly disagree	Disagree	Somewhat disagree	Somewhat agree	Agree	Strongly Agree
I feel safe after being vaccinated.	○	○	○	○	○	○
I can rely on vaccines to stop serious infectious diseases.	○	○	○	○	○	○
I feel protected after getting vaccinated.	○	○	○	○	○	○

Domain 2: Worries about unforeseen future events

	Strongly disagree	Disagree	Somewhat disagree	Somewhat agree	Agree	Strongly Agree
Although most vaccines appear to be safe, there may be problems that we have not yet discovered.	○	○	○	○	○	○
Vaccines can cause unforeseen problems in children.	○	○	○	○	○	○
I worry about the unknown effects of vaccines in the future.	○	○	○	○	○	○

Domain 3: Concerns about commercial profiteering

	Strongly disagree	Disagree	Somewhat disagree	Somewhat agree	Agree	Strongly Agree
Vaccines make a lot of money for pharmaceutical companies, but do not do much for regular people.	○	○	○	○	○	○
Authorities promote vaccination for financial gain not for people's health	○	○	○	○	○	○
Vaccination programs are a big con.	○	○	○	○	○	○

Domain 4: Preference for natural immunity

	Strongly disagree	Disagree	Somewhat disagree	Somewhat agree	Agree	Strongly Agree
Natural immunity lasts longer than a vaccination.	◯	◯	◯	◯	◯	◯
Natural exposure to viruses and germs gives the safest protection.	◯	◯	◯	◯	◯	◯
Being exposed to diseases naturally is safer for the immune system than being exposed through vaccination.	◯	◯	◯	◯	◯	◯

0 Have you been vaccinated against the coronavirus (COVID-19)?

◯ Yes, fully
◯ Yes, first dose and await second dose
◯ No
◯ Prefer not to answer

b Has the vaccine been offered to you?

◯ Yes
◯ No, please explain: ______
◯ Prefer not to answer

a Are you planning on getting the COVID-19 vaccine when it is available to you?

◯ Yes, please explain why: ______
◯ No, please explain why not: ______
◯ Undecided, please explain: ______
◯ Prefer not to answer

c Which vaccine did you receive?

◯ Pfizer
◯ Moderna
◯ Astra Zeneca
◯ Johnson & Johnson
◯ I don't know
◯ Other, specify: ______

i Date of first vaccine ________________________________

Date of second vaccine ______ ______

Which vaccine did you receive for your third dose (booster shot)?

◯ Pfizer
◯ Moderna
◯ Not applicable (no booster shot)

Date of third vaccine, if applicable
This is intended to collect the date of your first booster vaccine, if applicable. If you have not received a booster COVID-19 shot, please leave blank. ________________________________

Powered by REDCap

1 Where did you receive your vaccine?

◯ In my local community
◯ In a nearby community
◯ Other, please specify: ______
◯ Prefer not to answer

a Why did you choose that location?

☐ Location was convenient
☐ It was the only place offered
☐ It was the location I was told to go to
☐ Other, please specify: ______
☐ Prefer not to answer

Did you experience an adverse event(s) from the vaccine for which you sought medical attention?

◯ Yes ◯ No ◯ Prefer not to answer

b Did you experience any of the following adverse events?

☐ Allergic skin reactions (e.g. hives)
☐ Pain/redness/swelling at the injection site
☐ Other severe or unusual events
☐ Rash
☐ Fever along with another reportable event
☐ Large or sore lymph nodes (glands in your neck, armpits, groin)
☐ Numbness or tingling
☐ Severe vomiting/diarrhea
☐ Cellulitis (infection of skin near injection site)
☐ Severe vomiting/diarrhea
☐ Event managed as anaphylaxis (received epinephrine)
☐ Seizure
☐ Other, specify: ______
☐ None of the above

i Were you hospitalized?

◯ Yes ◯ No ◯ Prefer not to answer

2 Should the vaccine be made mandatory?

◯ Yes, please explain why: ______
◯ No, please explain why not: ______
◯ Undecided
◯ Prefer not to answer

3 Please show on the following scale your level of concern with the common side effects from the COVID-19 vaccine (e.g. pain/swelling at injection site, fatigue, fever, muscle ache, etc).

Not at all — Neutral — A very great concern

(Place a mark on the scale above)

Do you have any South Asian family members or friends with concerns about getting the COVID-19 vaccine?

◯ Yes ◯ No

4 Have you used any traditional, ancestral, or home remedies to increase your immunity against COVID-19?

◯ Yes
◯ No
◯ Prefer not to answer

a Where did you learn of these remedies?

☐ Social media
☐ Radio
☐ Friend
☐ Family
☐ Elder
☐ Healer
☐ Prefer not to answer

Powered by REDCap

Covid19 Assessment (5-10 minutes)

Please respond to the questions below in a way that best reflects your understanding of COVID-19, even if you have not experienced any COVID-19 symptoms yourself and you do not know anyone else that has been affected – your insights are valuable to us.

If you need to leave before you are finished these questions, please click "Save & Return Later" at the bottom of the screen. We will then send you an email link to continue with the survey.

If you have any questions about this survey, please email our study team at covid.community.on@phri.ca

---

Today ________________________________

---

1 Did you have any trouble finding information on COVID-19?
Examples:

What are the symptoms of COVID-19? What to do if you think you have COVID-19? Where do I go to get a test for COVID?

◯ Yes
◯ No
◯ Prefer not to answer

---

2 Have you ever been tested for COVID-19?

◯ Yes
◯ No
◯ Prefer not to answer

---

a How many times have you been tested? ______ ______
What was the date of the first test?

______
Year

/ ______
Month

You can access your COVID-19 test history here: https://covid19results.ehealthontario.ca:4443/agree

---

Information on accessing your COVID-19 test history is here: http://www.bccdc.ca/health-info/diseases-conditions/covid-19/testing/test-results

---

b What was the result of the first test?

◯ Negative (don't/didn't have COVID)
◯ Positive (do/did have COVID)
◯ Inconclusive
◯ Don't know
◯ Prefer not to answer

---

c Did you have any symptoms of COVID-19 when you had your first test?

◯ Yes
◯ No
◯ Don't know
◯ Prefer not to answer

i What symptoms did you have?

☐ Cough
☐ Fever
☐ Shortness of breath
☐ Chest pain
☐ Sore muscles
☐ Headache
☐ Sore throat
☐ Diarrhea
☐ Decreased sense of smell or taste
☐ Fatigue
☐ Delirious
☐ Other, specify: ______

---

ii Did your symptoms last more than 4 weeks?

◯ Yes
◯ No
◯ Prefer not to answer

How much are you currently affected in your everyday life by COVID-19?
Please indicate which one of the following statements applies to you most.

◯ I have no limitations in my everyday life and no symptoms, pain, depression or anxiety related to the infection.
◯ I have negligible limitation in my everyday life as I can perform all usual duties/activities, although I still have persistent symptoms, pain, depression or anxiety.
◯ I suffer from limitations in my everyday life as I occasionally need to avoid or reduce usual duties/activities or need to spread these over time due to symptoms, pain, depression or anxiety. I am, however, able to perform all activities without any assistance.
◯ I suffer from limitations in my everyday life as I am not able to perform all usual duties/activities due to symptoms, pain, depression or anxiety. I am, however, able to take care of myself without any assistance.
◯ I suffer from severe limitations in my everyday life: I am not able to take care of myself and therefore I am dependent on nursing care and/or assistance from another person due to symptoms, pain, depression or anxiety.

---

iii Do you still have symptoms?

◯ Yes
◯ No
◯ Prefer not to answer

---

3 Do you think you have had COVID-19?

◯ Yes
◯ No
◯ Prefer not to answer

---

a How do you know you had COVID-19?

☐ Online Symptom review
☐ I had symptoms of COVID-19
☐ Positive COVID-19 Nasal/throat test result
☐ Positive COVID-19 Blood test
☐ A Health care provider told me
☐ I was in contact with a confirmed case of COVID-19
☐ Other, please specify: ______
☐ Prefer not to answer

i What symptoms did you have?

☐ Cough
☐ Fever
☐ Shortness of breath
☐ Chest pain
☐ Sore muscles
☐ Headache
☐ Sore throat
☐ Diarrhea
☐ Decreased sense of smell or taste
☐ Fatigue
☐ Delirious
☐ Other, specify: ______

i What was the date you were diagnosed?

____

b What treatment did you receive?

☐ Self-isolation (Quarantine) at home or at an isolation facility
☐ Hospitalized ______
☐ Prefer not to answer

c Where do you think you might have contracted or been exposed to COVID-19?

☐ International travel
☐ Travel in Canada
☐ Travel away from my community
☐ At work
☐ Household
☐ Visiting family/friends
☐ Public gathering (eg. house parties, wedding events, funerals, religious services)
☐ Restaurant/Bars/Clubs
☐ Healthcare setting (at hospital, doctor's appointment etc.)
☐ Grocery/retail outing
☐ Gym/Sports activities
☐ School/daycare
☐ Other, please specify: ______
☐ I don't know where I was exposed
☐ Prefer not to answer

d Did you travel in the 6 months before your symptoms began?

◯ Yes
◯ No
◯ Don't know
◯ Prefer not to answer

e How many times have you been in a gathering with people outside your household in the 6 months before your symptoms began? ______ ______

4 How often you have done the following since March 2020?

		Never	Rarely	Occasionall y	Often	Always	N/A	Prefer not to answer
a	Worn a mask in public places	◯	◯	◯	◯	◯	◯	◯
b	Practiced physical distancing in public places	◯	◯	◯	◯	◯	◯	◯
c	Avoided crowded places/gatherings	◯	◯	◯	◯	◯	◯	◯
d	Avoided common greetings (such as a handshake or hug)	◯	◯	◯	◯	◯	◯	◯
e	Limited contact with people at higher risk (eg. An elderly relative)	◯	◯	◯	◯	◯	◯	◯
f	Self-isolated because you were diagnosed with COVID-19, were waiting for results, had symptoms (even mild) and you had been in contact with someone with confirmed or suspected COVID-19, or were directed by your public health authority	◯	◯	◯	◯	◯	◯	◯
g	Self-quarantined due to travel, close contact with a confirmed or suspected case of COVID-19, or as directed by public health authority, but did not show symptoms	◯	◯	◯	◯	◯	◯	◯

Demographics Part 2 (< 5 minutes)

If you need to leave before you are finished these questions, please click "Save & Return Later" at the bottom of the screen. We will then send you an email link to continue with the survey.

If you have any questions about this survey, please email our study team at covid.community@phri.ca

---

Today

____

---

1 How many people reside in your immediate household, including yourself?

Children under 18 years old ______ ______
Adults 18 to 59 years old ______
Adults 60 to 69 years old ______
Adults 70 to 79 years old ______
Adults 80+ years old ______

---

2 How many bedrooms are in your house? ______ ______

How many bathrooms are in your household?

____

How many bathrooms are in your household? ◯ Prefer not to answer

---

3 Do you have a doctor or nurse as a primary care provider?

◯ Yes
◯ No
◯ Don't know
◯ Prefer not to answer

---

4 Do you usually get a flu shot?

◯ Yes
◯ No
◯ Prefer not to answer

---

5 What is the nearest major intersection to where you live?

Nearest major intersection ______ ______

What are the first 3 characters of your postal code?

____

What is your FSA (Forward Sortation Area is the first 3 characters of your postal code) where you reside? ◯ Prefer not to answer

Covid19 Information (10-15 minutes)

If you need to leave before you are finished these questions, please click "Save & Return Later" at the bottom of the screen. We will then send you an email link to continue with the survey.

If you have any questions about this survey, please email our study team at covid.community.on@phri.ca

---

Today

____

---

1 Where do you primarily get your health information on COVID-19?

☐ WhatsApp or other instant messaging platforms
☐ Social Media platforms ______
☐ Traditional Media sources (TV news channels, newspapers)
☐ Local media and radio programs (e.g. Cina Radio)
☐ Friends and family
☐ Co-workers
☐ Religious Leaders
☐ Celebrities/Influencers
☐ My healthcare provider (e.g. family doctor, pharmacist, physiotherapist etc.)
☐ Provincial Public health bodies (website, officials, public health nurse, etc.)
☐ Other, specify: ______
☐ Prefer not to answer

Other Health Information Source

____

---

2 Please select your three most trusted information sources for COVID-19.
(If you don't use three different information sources for COVID-19, please rank the sources you do use, from most to least trusted.)

	Most trusted	Second most trusted	Third most trusted
WhatsApp or other instant messaging platforms	○	○	○
Facebook community groups/pages	○	○	○
Instagram	○	○	○
Tik Tok	○	○	○
Twitter	○	○	○
Other social media platform	○	○	○
Traditional media sources	○	○	○
Local media and radio programs	○	○	○
Friends and family	○	○	○
Co-workers	○	○	○
Religious leaders	○	○	○
Celebrities/Influencers	○	○	○
My health care provider	○	○	○

Powered by REDCap

Provincial Public health bodies	○	○	○
[cihinfnm]	○	○	○

3 For the following sources, please indicate why they are not one of your three most trusted sources for COVID-19 health information.

WhatsApp or other instant messaging platforms

- ☐ Messages are not delivered in my preferred language
- ☐ Unable to understand the information
- ☐ Not receiving "complete" information
- ☐ Not possible for me to act on that information due to work or family commitments
- ☐ I find the information to be judgmental and discriminatory against my group
- ☐ Other, please specify: ______
- ☐ Not sure
- ☐ Prefer not to answer

Facebook community groups/pages

- ☐ Messages are not delivered in my preferred language
- ☐ Unable to understand the information
- ☐ Not receiving "complete" information
- ☐ Not possible for me to act on that information due to work or family commitments
- ☐ I find the information to be judgmental and discriminatory against my group
- ☐ Other, please specify: ______
- ☐ Not sure
- ☐ Prefer not to answer

Instagram

- ☐ Messages are not delivered in my preferred language
- ☐ Unable to understand the information
- ☐ Not receiving "complete" information
- ☐ Not possible for me to act on that information due to work or family commitments
- ☐ I find the information to be judgmental and discriminatory against my group
- ☐ Other, please specify: ______
- ☐ Not sure
- ☐ Prefer not to answer

Tik Tok

- ☐ Messages are not delivered in my preferred language
- ☐ Unable to understand the information
- ☐ Not receiving "complete" information
- ☐ Not possible for me to act on that information due to work or family commitments
- ☐ I find the information to be judgmental and discriminatory against my group
- ☐ Other, please specify: ______
- ☐ Not sure
- ☐ Prefer not to answer

Powered by REDCap

Twitter

- ☐ Messages are not delivered in my preferred language
- ☐ Unable to understand the information
- ☐ Not receiving "complete" information
- ☐ Not possible for me to act on that information due to work or family commitments
- ☐ I find the information to be judgmental and discriminatory against my group
- ☐ Other, please specify: ______
- ☐ Not sure
- ☐ Prefer not to answer

Traditional media sources

- ☐ Messages are not delivered in my preferred language
- ☐ Unable to understand the information
- ☐ Not receiving "complete" information
- ☐ Not possible for me to act on that information due to work or family commitments
- ☐ I find the information to be judgmental and discriminatory against my group
- ☐ Other, please specify: ______
- ☐ Not sure
- ☐ Prefer not to answer

Local media and radio programs

- ☐ Messages are not delivered in my preferred language
- ☐ Unable to understand the information
- ☐ Not receiving "complete" information
- ☐ Not possible for me to act on that information due to work or family commitments
- ☐ I find the information to be judgmental and discriminatory against my group
- ☐ Other, please specify: ______
- ☐ Not sure
- ☐ Prefer not to answer

Friends and family

- ☐ Messages are not delivered in my preferred language
- ☐ Unable to understand the information
- ☐ Not receiving "complete" information
- ☐ Not possible for me to act on that information due to work or family commitments
- ☐ I find the information to be judgmental and discriminatory against my group
- ☐ Other, please specify: ______
- ☐ Not sure
- ☐ Prefer not to answer

Co-workers

- ☐ Messages are not delivered in my preferred language
- ☐ Unable to understand the information
- ☐ Not receiving "complete" information
- ☐ Not possible for me to act on that information due to work or family commitments
- ☐ I find the information to be judgmental and discriminatory against my group
- ☐ Other, please specify: ______
- ☐ Not sure
- ☐ Prefer not to answer

Powered by REDCap

Religious leaders

- ☐ Messages are not delivered in my preferred language
- ☐ Unable to understand the information
- ☐ Not receiving "complete" information
- ☐ Not possible for me to act on that information due to work or family commitments
- ☐ I find the information to be judgmental and discriminatory against my group
- ☐ Other, please specify: ______
- ☐ Not sure
- ☐ Prefer not to answer

Celebrities or Influencers

- ☐ Messages are not delivered in my preferred language
- ☐ Unable to understand the information
- ☐ Not receiving "complete" information
- ☐ Not possible for me to act on that information due to work or family commitments
- ☐ I find the information to be judgmental and discriminatory against my group
- ☐ Other, please specify: ______
- ☐ Not sure
- ☐ Prefer not to answer

My health care provider

- ☐ Messages are not delivered in my preferred language
- ☐ Unable to understand the information
- ☐ Not receiving "complete" information
- ☐ Not possible for me to act on that information due to work or family commitments
- ☐ I find the information to be judgmental and discriminatory against my group
- ☐ Other, please specify: ______
- ☐ Not sure
- ☐ Prefer not to answer

Public health authorities

- ☐ Messages are not delivered in my preferred language
- ☐ Unable to understand the information
- ☐ Not receiving "complete" information
- ☐ Not possible for me to act on that information due to work or family commitments
- ☐ I find the information to be judgmental and discriminatory against my group
- ☐ Other, please specify: ______
- ☐ Not sure
- ☐ Prefer not to answer

[cihinfnm]

- ☐ Messages are not delivered in my preferred language
- ☐ Unable to understand the information
- ☐ Not receiving "complete" information
- ☐ Not possible for me to act on that information due to work or family commitments
- ☐ I find the information to be judgmental and discriminatory against my group
- ☐ Other, please specify: ______
- ☐ Not sure
- ☐ Prefer not to answer

---

4 For the following statements, please indicate how strongly you agree or disagree.

Powered by REDCap

		Strongly Disagree	Disagree	Neutral	Agree	Strongly Agree	Prefer not to answer
a	COVID-19 poses a major threat to our community	○	○	○	○	○	○
b	The situation around COVID-19 is overexaggerated/overblown	○	○	○	○	○	○
c	I am at a high risk from COVID-19 because of my location	○	○	○	○	○	○
d	I am at high risk from COVID-19 because of my housing situation	○	○	○	○	○	○
e	I am at a high risk from COVID-19 because of my profession or my work	○	○	○	○	○	○
f	I am at a high risk from COVID-19 because of my lifestyle (socializing or working in a crowded place)	○	○	○	○	○	○
g	If I get exposed to/contract COVID-19, I am likely to have serious symptoms because of age, and/or pre-existing conditions	○	○	○	○	○	○
h	If I get exposed to/contract COVID-19, I am likely to need hospitalization because of my age, and/or pre-existing conditions	○	○	○	○	○	○

Mental And Emotional Impact Of Covid19 (5-10 minutes)

If you need to leave before you are finished these questions, please click "Save & Return Later" at the bottom of the screen. We will then send you an email link to continue with the survey.

If you have any questions about this survey, please email our study team at covid.community.on@phri.ca

Today ________________________

1 The questions below are meant to find out how you have been feeling since the COVID-19 was declared as a pandemic (March 2020).

Since the onset of the Pandemic, have you experienced the following?

		N/A	Never	Rarely	Sometimes	Often	Always
a	Racism in the community	◯	◯	◯	◯	◯	◯
b	Burnout from your home life (e.g. caring for family members, home schooling)	◯	◯	◯	◯	◯	◯
c	Racism in the workplace	◯	◯	◯	◯	◯	◯
d	Feeling stigmatized for working in a factory, processing centre, or as a health care worker	◯	◯	◯	◯	◯	◯
e	Feelings of pressure to continue to go to work each day	◯	◯	◯	◯	◯	◯
f	Health benefits available that cover you for sick days	◯	◯	◯	◯	◯	◯
g	Burnout from your work life	◯	◯	◯	◯	◯	◯

Language for translation
- ◯ Bengali
- ◯ English
- ◯ Gujarati
- ◯ Hindi
- ◯ Punjabi
- ◯ Tamil
- ◯ Urdu

2 In the past 2 weeks, how often have you been bothered by the following?

		Not at all	Several days	More than half the days	Nearly every day
a	Little interest or pleasure in doing things	◯	◯	◯	◯
b	Feeling down, depressed or hopeless	◯	◯	◯	◯
c	Trouble falling or staying asleep, or sleeping too much	◯	◯	◯	◯
d					

	Feeling tired or having little energy	◯	◯	◯	◯
e	Poor appetite or overeating	◯	◯	◯	◯
f	Feeling bad about yourself- or that you are a failure or have let yourself or your family down	◯	◯	◯	◯
g	Trouble concentrating on things, such as reading the newspaper or watching television	◯	◯	◯	◯
h	Moving or speaking so slowly that other people could have noticed. Or the opposite- being so fidgety or restless that you have been moving around a lot more than usual	◯	◯	◯	◯
i	Thoughts that you would be better off dead, or of hurting yourself	◯	◯	◯	◯

3 In the past 2 weeks, how often have you been bothered by the following?

		Not at all	Several days	More than half the days	Nearly every day
a	Feeling nervous, anxious, or on edge	◯	◯	◯	◯
b	Not being able to stop or control worrying	◯	◯	◯	◯
c	Worrying too much about different things	◯	◯	◯	◯
d	Trouble relaxing	◯	◯	◯	◯
e	Being so restless that it's hard to sit still	◯	◯	◯	◯
f	Becoming easily annoyed or irritable	◯	◯	◯	◯
g	Feeling afraid as if something awful might happen	◯	◯	◯	◯

3 How has COVID-19 affected your relationships?
My relationship with....

		N/A	Is closer or easier	Is about the same	Is more distant or strained	Prefer not to answer
a	My intimate partner	◯	◯	◯	◯	◯
b	Other family members (excluding intimate partner)	◯	◯	◯	◯	◯
c						

Powered by REDCap

	Friends	◯	◯	◯	◯	◯
d	Neighbours	◯	◯	◯	◯	◯
e	People I don't know but are in my community	◯	◯	◯	◯	◯
f	Work colleagues	◯	◯	◯	◯	◯

How has the pandemic affected your mental health?

◯ Improved
◯ A bit better
◯ No change
◯ Somewhat worse
◯ Substantially worse

Please describe:

◯ Depression
◯ Anxiety
◯ Other, please specify: ______

Please describe the impact the pandemic has had on you.

Cardiovascular Risk Factors

If you need to leave before you are finished these questions, please click "Save & Return Later" at the bottom of the screen. We will then send you an email link to continue with the survey.

If you have any questions about this survey, please contact our study team.

Today

____

---

1 Have you ever smoked any tobacco products (cigarettes)? ◯ Yes ◯ No

---

a Have you smoked in the last 12 months? ◯ Yes ◯ No

---

b What was your average smoking pattern over the last 12 months?

I smoked on average ______ cigarettes ______

Average number of cigarettes smoked in a day

____

---

2 Over the past 12 months, what has been your typical exposure to other people's tobacco smoke?

◯ Less than one hour of exposure in a week
◯ One or more hours of exposure in a week

---

3 Do you have diabetes mellitus? ◯ Yes ◯ No ◯ Unsure

---

4 Do you have high blood pressure? ◯ Yes ◯ No ◯ Unsure

---

5 Have either of your biological parents had a heart attack? ◯ Yes ◯ No ◯ Unsure

---

6 How often have you felt work or home life stress in the last year?

◯ Never or some periods
◯ Several periods of stress or permanent stress

---

7 During the last year, was there ever a time when you felt sad, blue, or depressed for two weeks or more in a row? ◯ Yes ◯ No

---

8 Do you eat salty food or snacks one or more times a day? ◯ Yes ◯ No

---

9 Do you eat deep fried foods or snacks or fast foods 3 or more times a week? ◯ Yes ◯ No

---

10 Do you eat fruit one or more times daily? ◯ Yes ◯ No

---

11 Do you eat vegetables one or more times daily? ◯ Yes ◯ No

---

12 Do you eat meat or poultry 2 or more times daily? ◯ Yes ◯ No

13 How active are you during your leisure time?

◯ I am mainly sedentary or perform mild exercise (requiring minimal effort)
◯ I perform moderate or strenuous physical activity

Waist to hip ratio

____

Powered by REDCap

Physical Measures (5 minutes)

Please complete the physical measurements below. All measurements should be taken in light clothing (no shoes when measuring your height).

If you need to leave before you are finished these questions, please click "Save & Return Later" at the bottom of the screen. We will then send you an email link to continue with the survey.

If you have any questions about this survey, please email our study team at covid.community.on@phri.ca

---

Today ________________________________

Who is reporting the physical measures?

◯ Participant
◯ Vaccine site staff
◯ Not collected

Weight: ______ ______
Height: ____________ ______ ______
When recording your height, please select the units before you enter your height

---

TANITA % body fat ______

TANITA % body fat ______

How to take waist and hip measurements:

Use a tape measure. Take measurement with a light layer on. If you can, have someone help you with the measurements Make sure the tape measure is horizontal the whole way around Waist: Take the measurement at the smallest horizontal diameter between your ribs and hips Hip: Take the measurement where your hip meets your thigh (often the biggest measurement of your buttocks)

Waist measurement: ______ ______
Hip measurement: ______ ______

Second Vaccine Update

Please complete the survey below to update us on your new second vaccine appointment.

Thank you!

StudyId

____

Today

____

Original date of the second vaccine

____

Has the date for your second COVID-19 vaccine changed?
Your original scheduled vaccine date was [v2olddt]

○ Yes ○ No

When is your new appointment for the second dose?

____

Powered by REDCap

First Visit Short Questionnaire

This is an abbreviated survey, with the essential questions that you have not yet completed. It should take somewhere between < 1 and 10 minutes to complete.

If you have any questions about this survey or any other aspects of the study, please contact the study team at [studyemail].

Vaccination status

Date of Phone Survey ________________________________

Person completing phone survey

____

Have you been vaccinated against the coronavirus (COVID-19)?

◯ Yes, fully
◯ Yes, first dose and await second dose
◯ No
◯ Prefer not to answer

Has the vaccine been offered to you?

◯ Yes
◯ No, please explain: ______
◯ Prefer not to answer

Which vaccine did you receive?

◯ Pfizer
◯ Moderna
◯ Astra Zeneca
◯ Johnson & Johnson
◯ I don't know
◯ Other, specify: ______

Date of first vaccine ________________________________

Date of second vaccine ______ ______

Original date of the second vaccine ________________________________

Has the date for your second COVID-19 vaccine changed? Your original scheduled vaccine date was [psolddt] ◯ Yes ◯ No

When is your new appointment for the second dose? ________________________________

Powered by REDCap

Have you received a booster shot for the COVID-19 vaccine?

◯ Yes
◯ No
◯ Prefer not to answer

Which vaccine did you receive for your booster shot?

◯ Pfizer
◯ Moderna

Date of booster vaccine

____

Did you experience an adverse event(s) from the vaccine for which you sought medical attention?

◯ Yes ◯ No ◯ Prefer not to answer

Powered by REDCap

Vaccine Confidence

For the next set of questions, please indicate how strongly you agree or disagree with each statement.

Powered by REDCap

Domain 1: Mistrust of vaccine benefit

	Strongly disagree	Disagree	Somewhat disagree	Somewhat agree	Agree	Strongly Agree
I feel safe after being vaccinated.	◯	◯	◯	◯	◯	◯
I can rely on vaccines to stop serious infectious diseases.	◯	◯	◯	◯	◯	◯
I feel protected after getting vaccinated.	◯	◯	◯	◯	◯	◯

Powered by REDCap

Domain 2: Worries about unforeseen future events

	Strongly disagree	Disagree	Somewhat disagree	Somewhat agree	Agree	Strongly Agree
Although most vaccines appear to be safe, there may be problems that we have not yet discovered.	◯	◯	◯	◯	◯	◯
Vaccines can cause unforeseen problems in children.	◯	◯	◯	◯	◯	◯
I worry about the unknown effects of vaccines in the future.	◯	◯	◯	◯	◯	◯

Domain 3: Concerns about commercial profiteering

	Strongly disagree	Disagree	Somewhat disagree	Somewhat agree	Agree	Strongly Agree
Vaccines make a lot of money for pharmaceutical companies, but do not do much for regular people.	○	○	○	○	○	○
Authorities promote vaccination for financial gain not for people's health	○	○	○	○	○	○
Vaccination programs are a big con.	○	○	○	○	○	○

Powered by REDCap

Domain 4: Preference for natural immunity

	Strongly disagree	Disagree	Somewhat disagree	Somewhat agree	Agree	Strongly Agree
Natural immunity lasts longer than a vaccination.	○	○	○	○	○	○
Natural exposure to viruses and germs gives the safest protection.	○	○	○	○	○	○
Being exposed to diseases naturally is safer for the immune system than being exposed through vaccination.	○	○	○	○	○	○

Powered by REDCap

COVID-19 history

Do you think you have had COVID-19?

◯ Yes
◯ No
◯ Prefer not to answer

How do you know you had COVID-19?

☐ Online Symptom review
☐ I had symptoms of COVID-19
☐ Positive COVID-19 Nasal/throat test result
☐ Positive COVID-19 Blood test
☐ A Health care provider told me
☐ I was in contact with a confirmed case of COVID-19
☐ Other, please specify: ______
☐ Prefer not to answer

What was the date you were diagnosed?

____

What treatment did you receive?

☐ Self-isolation (Quarantine) at home or at an isolation facility
☐ Hospitalized ______
☐ Prefer not to answer

Other health history

Have you ever...

Had a heart attack?	◯ Yes ◯ No ◯ Unsure
Had angioplasty, stent in your heart, or heart bypass surgery?	◯ Yes ◯ No ◯ Unsure
Suffered a stroke?	◯ Yes ◯ No ◯ Unsure
Been diagnosed with cancer?	◯ Yes ◯ No ◯ Unsure
Do you have a chronic medical condition that requires you to take medication(s) everyday?	◯ Yes ◯ No ◯ Prefer not to answer

Please specify which condition(s) require you to take medication everyday.

☐ Diabetes
☐ High blood pressure
☐ Heart disease or Stroke
☐ Arthritis
☐ Chronic Lung Disease
☐ Cancer
☐ Other, specify: ______

Demographics & other contextual information

What is your mother tongue?

☐ Bengali
☐ English
☐ Gujarati
☐ Hindi
☐ Punjabi
☐ Tamil
☐ Urdu
☐ Other, specify: ______
☐ Prefer not to answer

What is your religious affiliation?

☐ Buddhism
☐ Christianity
☐ Hinduism
☐ Islam
☐ Jainism
☐ Sikhism
☐ Other, specify: ______
☐ Prefer not to answer

Were you born in Canada?

◯ Yes
◯ No
◯ Prefer not to answer

What is the highest level of education you have completed?

◯ Less than high school graduation
◯ High school graduate
◯ Trade certificate, vocational school, or apprenticeship training
◯ Non-university certificate or diploma from a community college, CEFEP
◯ University bachelor's degree
◯ University graduate degree (e.g. masters or doctorate)
◯ Prefer not to answer

What is your marital status?

◯ Never Married
◯ Common law/Living with partner
◯ Currently married
◯ Divorced/Separated
◯ Widowed
◯ Prefer not to answer

What is your current employment status?

◯ Employed, please specify where you work: ______
◯ Unemployed
◯ Retired
◯ Temporarily laid off due to COVID-19
◯ Permanently laid off due to COVID-19
◯ Prefer not to answer

Powered by REDCap

What is your best estimate of the total income of ALL household members from ALL sources in the past 12 months (before taxes)?

◯ $0 - 19,999
◯ $20,000 - 39,999
◯ $40,000 - 59,999
◯ $60,000 - 79,999
◯ $80,000 - 99,999
◯ $100,000 and above
◯ Prefer not to answer

What is your country of birth?

◯ India
◯ Pakistan
◯ Sri Lanka
◯ Bangladesh
◯ Guyana
◯ Trinidad
◯ Uganda
◯ Tanzania
◯ South Africa
◯ United Arab Emirates
◯ Other, specify: ______
◯ Prefer not to answer

How many years have you lived in Canada?

◯ < 5 years
◯ 5 - 10 years
◯ > 10 years
◯ Prefer not to answer

How many people reside in your immediate household, including yourself?

Children under 18 years old ______ ______
Adults 18 to 59 years old ______
Adults 60 to 69 years old ______
Adults 70 to 79 years old ______
Adults 80+ years old ______

Have you ever smoked any tobacco products (cigarettes)? ◯ Yes ◯ No

Have you smoked in the last 12 months? ◯ Yes ◯ No

What was your average smoking pattern over the last 12 months?

I smoked on average ______ cigarettes ______

Average number of cigarettes smoked in a day

____
